# Overexpression of CXCL14 Alleviates Ventilator-Induced Lung Injury through the Downregulation of PKM2-Mediated Cytokine Production

**DOI:** 10.1155/2020/7650978

**Published:** 2020-07-23

**Authors:** Rui Zhu, Yan-Qing Lei, Dong-Chi Zhao

**Affiliations:** ^1^Department of Pediatrics, Zhongnan Hospital of Wuhan University, 169 Donghu Road, Wuhan, Hubei 430071, China; ^2^Department of Emergency, Xiangyang No.1 People's Hospital, Hubei University of Medicine, 15 Jiefang Road, Xiangyang, Hubei 441000, China

## Abstract

Ventilator-induced lung injury (VILI) is one of the most common complications of mechanical ventilation (MV), which strongly impacts the outcome of ventilated patients. Current evidences indicated that inflammation is a major contributor to the pathogenesis of VILI. Our results showed that MV induced excessive proinflammatory cytokine productions together with decreased CXCL14 and increased PKM2 expressions in injured lungs. In addition, CXCL14 overexpression downregulated PKM2 expression and attenuated VILI with reduced inflammation. Moreover, the overexpression of PKM2 markedly diminished the protective effects of CXCL14 against VILI as reflected by worsened morphology and increased cytokine production, whereas PKM2 knockdown decreased cytokine production and attenuated VILI. Collectively, these results suggested that CXCL14 overexpression attenuates VILI through the downregulation of PKM2-mediated proinflammatory cytokine production.

## 1. Introduction

Numerous evidences have demonstrated that mechanical ventilation (MV) causes damages to lungs in both animals and humans, which is defined as ventilator-induced lung injury (VILI) [1]. VILI is the most common complication of using MV to treat acute respiratory distress syndrome (ARDS) [1], although MV can be injurious to the lungs and other organs simultaneously [2–4]. Ultimately, VILI is caused by impaired gas exchange due to collapse of the alveoli, or the alveolar flooding, leading to respiratory failure in critically ill patients [5]. Although our understanding of its pathophysiology is incomplete, it is commonly accepted that inflammation is one of the major contributors to the pathogenesis of VILI [6].

Pyruvate kinase M2 (PKM2), the M2 isoform of pyruvate kinase, catalyzes the final and rate-limiting reaction in the glycolytic pathway. PKM2 is expressed in few types of proliferating normal cells but overexpressed in cancer cells and activated immune cells [7]. It has been reported that PKM2 could promote interleukin-1beta (IL-1*β*) release in lipopolysaccharide- (LPS-) stimulated macrophages [8]. In addition, the accumulation of nuclear PKM2 is able to attenuate LPS-induced inflammatory response in injured lungs [9]. However, to the best of our knowledge, the roles of PKM2 in VILI have not been well studied.

CXCL14 is a relatively novel non-ELR (glutamic acid-leucine-arginine) chemokine with broad biological activities [10]. CXCL14 was initially identified from kidney and breast cells and has been reported to be widely expressed in normal tissue [10, 11]. The mechanisms governing the expression of CXCL14 and CXCL14-mediated functions are incompletely understood. It has been reported that CXCL14 is involved in immunity and inflammatory responses [12]. Frick et al. and Maerki et al. revealed that the expression of CXCL14 could be inhibited by TNF-*α* and lipopolysaccharide (LPS) [11, 13]. Recently, laboratory evidences demonstrated that CXCL14 plays a potential role in modulating inflammation and immunological responses [14]. However, whether CXCL14 is involved in the pathogenesis of VILI has not been reported. In the present study, we aim to investigate the roles of PKM2 and CXCL14 in VILI.

## 2. Materials and Methods

### 2.1. Animals

Adult male C57BL/6 mice were purchased from the Hubei Provincial Center for Disease Control and Prevention (Wuhan, Hubei, China). CXCL14 transgenic (CXCL14-Tg) mice were purchased from Cyagen (Suzhou, China). Animals were kept in individual cages under standard conditions. All animal experiments were approved (No. zn2018019) and were performed in the animal laboratory following the Guidelines of Animal Use and Care.

### 2.2. Study Design

*Experiment 1*. In order to investigate the roles of CXCL14 in the pathogenesis of VILI, animals were randomly assigned into the following groups: (1) a control group (*n* = 10): animals received sham operation without ventilation; (2) a MV group (*n* = 10): wild-type mice were mechanically ventilated for 18 hours under anesthesia; (3) a CXCL14-Tg group (*n* = 10): CXCL14-Tg mice received sham operation without ventilation; and (4) a CXCL14-Tg+MV group (*n* = 10): CXCL14-Tg mice were mechanically ventilated for 18 hours.

*Experiment 2*. This part was designed to determine the roles of PKM2 in the development of VILI and the association between CXCL14 and PKM2. Animals were randomly assigned into the following groups: (1) a MV+Sh-PKM2 group (*n* = 10): animals received pulmonary transfection of AAV5-Sh-PKM2 and then underwent ventilation for 18 hours; (2) a MV+Sh-Scram group (*n* = 10): animals received transfection of AAV5-Sh-Scram and then underwent 18 hours of MV; (3) a MV+PKM2 group (*n* = 10): animals were ventilated for 18 hours after pulmonary transfection of AAV5-PKM2; and (4) a CXCL14-Tg+PKM2+MV group (*n* = 10): CXCL14-Tg mice were given AAV5-PKM2 transfection and received MV for 18 hours.

### 2.3. Ventilator-Induced Lung Injury Model

In order to establish the VILI model, animals were mechanically ventilated for 18 hours. In detail, animals were anesthetized with intraperitoneal injection of sodium pentobarbital (50 mg/kg body weight). The right jugular vein was infused with sodium pentobarbital at a rate of 10 mg/kg/h for the anesthesia maintenance. Then, animals were tracheostomized and connected to a small animal ventilator (VentElite, Harvard Apparatus; Cambridge, MA, USA) using room air and a volume-controlled setting. VILI was achieved with the following ventilator parameters: respiration rate (RR) = 70 breaths/min; tidal volume (*V*_T_) = 20 ml/kg; and positive end-expiratory pressure (PEEP) = 0 cm H_2_O. Animals were sacrificed by blood dropping at the end of ventilation for sample collections. Specifically, the bronchoalveolar lavage fluid (BALF) was obtained from three bronchoalveolar lavages in the upper part of the trachea by using 5 ml of PBS.

### 2.4. *In Vivo* PKM2 Knockdown and Overexpression

Adeno-associated viral vectors carrying the specific short hairpin RNA for PKM2 (AAV5-Sh-PKM2, Santa Cruz Biotechnology, USA), the control sequence (AAV5-Sh-Scram, Guangzhou Ribobio Co., China), and the recombinant AAV5-PKM2 vectors were intratracheally injected into the lung tissues as previously described [15]. The AAV5-PKM2 vectors were packaged and purified by Wuhan Yuancheng Chemical Reagent Co., Ltd. (Wuhan, China). The effects of transfections were supported by qRT-PCR and Western blots.

### 2.5. Measurements of Cytokines

The expressions of proinflammatory cytokines including tumor necrosis factor-alpha (TNF-*α*), interleukin- (IL-) 1beta (IL-1*β*), and IL-6 were determined using commercial enzyme-linked immunosorbent assay (ELISA) kits according to the manufacturer's protocols (Abcam, Shanghai, China).

### 2.6. Histological Analysis of Injured Lungs

Specimens were fixed in 10% formalin, embedded in paraffin, cut into sections 4 *μ*m in thickness, and stained with hematoxylin-eosin (H&E) following the standard protocol. Lung injury index was calculated as previously described [16]. In detail, pulmonary injuries were scored from 0 to 4 based on the degrees of hemorrhage, edema, and infiltration of inflammatory cells and the histopathological changes: 0 score: no visible injury; 1 score: modest injury; 2 score: intermediate injury; 3 score: widespread injury; and 4 score: severe injury (such as marked congestion and interstitial edema with neutrophilic infiltrate).

### 2.7. Lung Wet/Dry Ratio

At the end of experiments, the middle lobe of the left lungs was removed and weighted to determine the wet weight (ww). Then, tissues were dried at 80°C for 48 hours for the stable dry weight (dw). The wet/dry ratio was calculated as (ww − dw)/dw.

### 2.8. Western Blots

Proteins were extracted in lysis buffer, and concentrations were determined using a BCA protein assay kit (Beyotime, China). Proteins were then separated by SDS-PAGE and transferred to membranes. Then, membranes were incubated with primary antibody against PKM2 and CXCL14 (Abcam) at 4°C overnight and were probed with HRP-labeled secondary antibody. The membranes were visualized using an enhanced chemiluminescence system (Kodak, Rochester, USA). GAPDH was used as a loading control.

### 2.9. RNA Extraction and Real-Time PCR

RNA was isolated from pulmonary tissues with TRIzol reagent. Total RNA concentration was assessed, and RT was performed using SuperScript II Reverse Transcriptase (Invitrogen) according to manufacturer's instructions. PCRs were conducted using the following primers: CXCL14: 5′-GCTTCCAGATGTGAGATCCAG-3′ and 5′-AGTAGACTGAGTTCCTCTA-3′; PKM2: 5′-TCGCATGCAGCACCTGATT-3′ and 5′-CCTCGAATAGCTGCAAGTGGTA-3′.

### 2.10. Statistical Analysis

Data were expressed as number, median, or mean ± SD. Comparison of means between groups was determined using ANOVA followed by Student's *t*-tests. Correlation analyses were performed by Pearson correlation analysis. Lung injury index was compared using the Steel-Dwass test followed by the Kruskal-Wallis test. Two-tailed *p* values less than 0.05 were considered significant. Statistical analysis was performed using the SPSS 18.0 software package (SPSS Inc., Chicago, USA).

## 3. Results

### 3.1. CXCL14 Expressions Were Negatively but PKM2 Expressions Were Positively Associated with Cytokine Productions in Injured Lungs after MV

**Figure 1:**
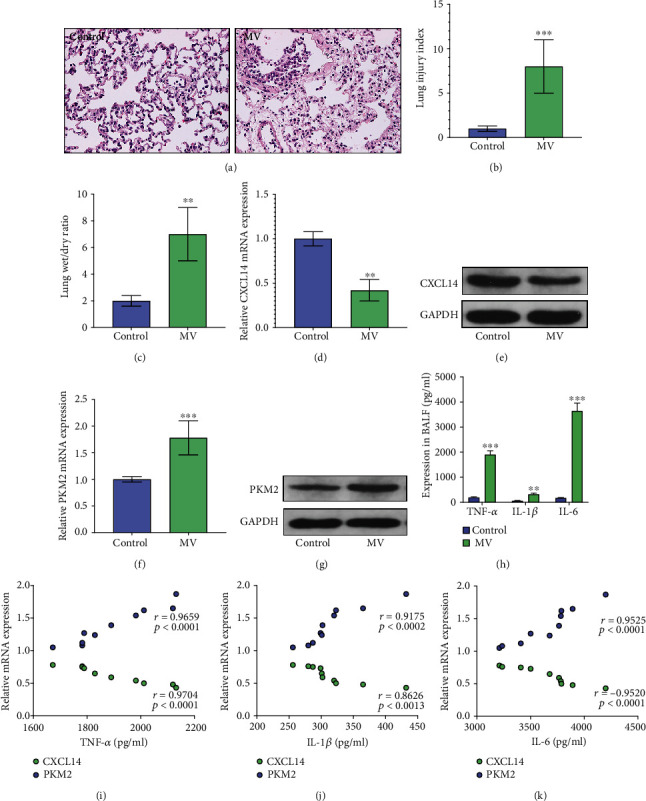
MV induced excessive production of proinflammatory cytokines together with downregulated CXCL14 and upregulated PKM2 expressions in the lungs. H&E stain (a) demonstrated apparent histological damages in the lungs after MV. In addition, lung injury index (b) and wet/dry ratio (c) were significantly increased in the MV group as compared with the control group. RT-PCR (d) and Western blots (e) demonstrated that pulmonary CXCL14 were significantly downregulated by MV, whereas expressions of PKM2 were significantly increased after MV (f, g). ELISA assays (h) showed that proinflammatory cytokines including TNF-*α*, IL-1*β*, and IL-6 were significantly increased in the MV group as compared with the control group. Moreover, correlation analysis (i–k) indicated that CXCL14 expressions were negatively, whereas PKM2 were positively associated with proinflammatory cytokines. ^∗∗^*p* < 0.01, ^∗∗∗^*p* < 0.0001 vs. the control group.

After 18 hours of MV, we observed apparent lung injury in mice as reflected by morphological damages and increased lung injury index and wet/dry ratio (Figures 1(a)–1(c)). In addition, our results demonstrated that pulmonary expressions of CXCL14 in the MV group were significantly lower than those in the control group (Figures 1(d) and 1(e)), whereas PKM2 expressions were significantly increased in the MV group as compared with the control group (*p* < 0.05, respectively) (Figures 1(f) and 1(g)). Moreover, MV induced excessive expressions of proinflammatory cytokines including TNF-*α*, IL-1*β*, and IL-6 in the injured lungs (Figure 1(h)), and the correlation analysis indicated that proinflammatory cytokines including TNF-*α*, IL-1*β*, and IL-6 were negatively associated with pulmonary CXCL14 expressions; however, pulmonary PKM2 expressions were positively associated with proinflammatory cytokine productions (Figures 1(i)–1(k)).

### 3.2. Overexpression of CXCL14 Attenuated VILI with Downregulated PKM2 Expression and Cytokine Production

**Figure 2:**
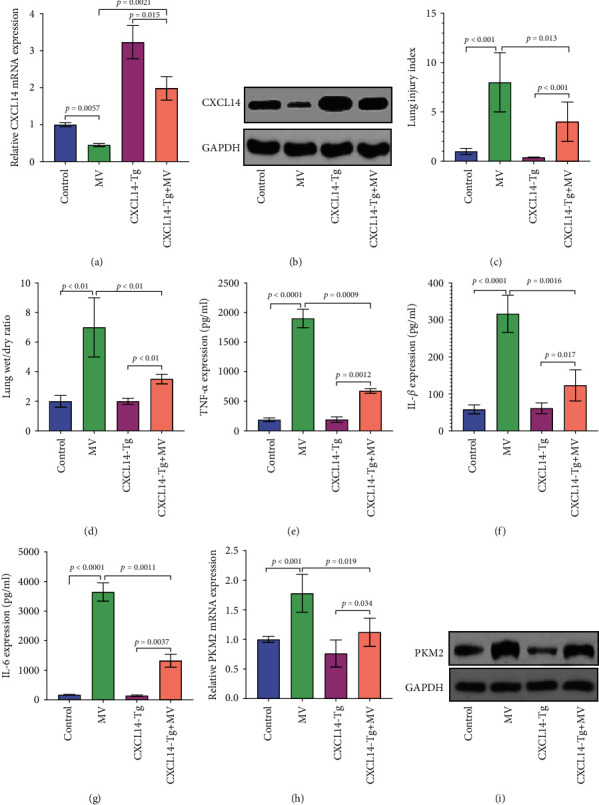
CXCL14 overexpression attenuated VILI and downregulated pulmonary PKM2 and cytokine expressions. RT-PCR (a) and Western blots (b) showed increased pulmonary expressions of CXCL14 in the CXCL14-Tg mice as compared with the wild-type mice (control and MV groups), indicating an overexpression of CXCL14. CXCL14-Tg mice showed improved pulmonary morphology as reflected by decreased lung injury index (c) and wet/dry ratio (d) as compared with wild-type mice (MV group). Moreover, proinflammatory cytokines including TNF-*α* (e), IL-1*β* (f), and IL-6 (g) were markedly decreased in the CXCL14-Tg+MV group as compared with the MV group. Moreover, RT-PCR (h) and Western blots (i) showed significantly lower expressions of PKM2 in the CXCL14-Tg mice than in the wild-type mice after MV.

RT-PCR and Western blots showed that CXCL14 mRNA and protein expressions in CXCL14-Tg mice were significantly higher than those in wild-type mice, indicating an overexpression of CXCL14 (Figures 2(a) and 2(b)). Next, our results suggested that CXCL14 overexpression attenuated VILI as reflected by lower lung injury index and decreased wet/dry ratio (Figures 2(c) and 2(d)). In addition, proinflammatory cytokines (TNF-*α*, IL-1*β*, and IL-6) at the end of MV were significantly lower in the CXCL14-Tg+MV group than in the MV group (*p* < 0.05, respectively) (Figures 2(e)–2(g)). Importantly, pulmonary expressions of PKM2 were also significantly decreased in the CXCL14-Tg+MV group as compared with the MV group (Figures 2(h) and 2(i)). Together, these results indicated that CXCL14 overexpression attenuates VILI with downregulated PKM2 and inflammatory cytokine production.

### 3.3. PKM2 Knockdown Attenuated VILI with Downregulated Cytokine Expressions

**Figure 3:**
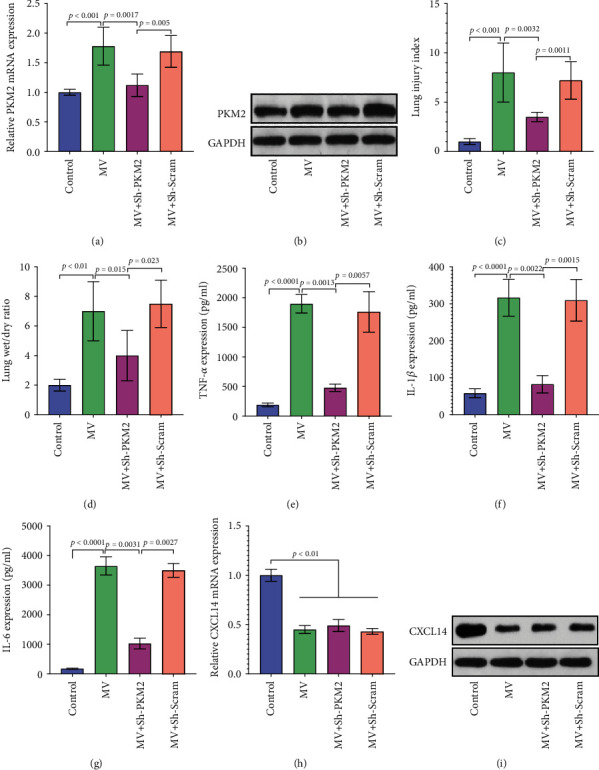
PKM2 knockdown attenuated VILI with downregulated cytokine productions. RT-PCR (a) and Western blots (b) identified a significant lower pulmonary expression of PKM2 in the MV+Sh-PKM2 group than in the MV group at 48 hours after transfection. Importantly, VILI has been markedly attenuated by PKM2 knockdown. In detail, decreased lung injury index (c) and lower wet/dry ratio (d) have been observed in the MV+Sh-PKM2 group as compared with the MV group. Moreover, proinflammatory cytokines including TNF-*α* (e), IL-1*β* (f), and IL-6 (g) were markedly decreased in the MV+Sh-PKM2 group than in the MV group. However, pulmonary expression levels of CXCL14 mRNA (h) and protein (i) remained unchanged after AAV5-Sh-PKM2 or AAV5-Sh-Scram transfections.

As seen in Figures 3(a) and 3(b), RT-PCR and Western blots demonstrated that pulmonary PKM2 expressions were significantly decreased in the MV+Sh-PKM2 group than in the MV and MV+Sh-Scram groups, indicating an efficient knockdown of PKM2. In addition, PKM2 knockdown attenuated VILI as reflected by lower lung injury index and decreased wet/dry ratio (Figures 3(c) and 3(d)). Importantly, ELISA assays demonstrated that the expressions of TNF-*α*, IL-1*β*, and IL-6 were significantly decreased in the MV+Sh-PKM2 group as compared with the MV and MV+Sh-Scram groups (*p* < 0.05, respectively) (Figures 3(e)–3(g)). However, pulmonary expressions of CXCL14 remained unchanged after AAV5-Sh-PKM2 or AAV5-Sh-Scram transfections (Figures 3(h) and 3(i)). Together, these results suggested that PKM2 knockdown was able to attenuate VILI through the downregulation of proinflammatory cytokines.

### 3.4. Overexpression of PKM2 Abolished the Protective Effects of CXCL14 Overexpression on VILI in Mice

**Figure 4:**
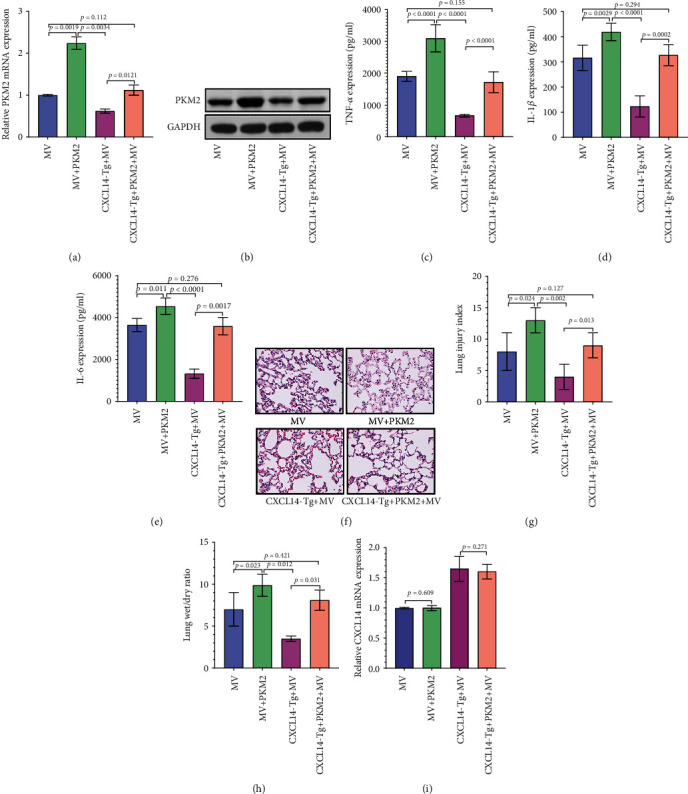
Overexpression of PKM2 abolished the protective effects of CXCL14 against VILI. RT-PCR (a) and Western blots (b) identified an overexpression of PKM2 in injured lungs at 48 hours after transfection. Importantly, the protective effects of CXCL14 against VILI had been markedly diminished by PKM2 overexpression. In detail, increased expressions of proinflammatory cytokines including TNF-*α* (c), IL-1*β* (d), and IL-6 (e), worsened morphology (f), increased lung injury index (g), and higher wet/dry ratio (h) has been observed in the CXCL14-Tg+PKM2 group as compared with the CXCL14-Tg+MV group. However, no significant differences in CXCL14 mRNA (i) expressions have been found between the CXCL14-Tg+MV and CXCL14-Tg+PKM2+MV groups.

Next, we further determined the roles of PKM2 in the development of VILI. Our results showed that PKM2 overexpression (Figures 4(a) and 4(b)) accelerated pulmonary expressions of proinflammatory cytokines in both CXCL14-Tg and wild-type mice after MV (Figures 4(c)–4(e)). Histological analysis showed that PKM2 overexpression worsened histopathological damages (Figure 4(f)) with increased lung injury index (Figure 4(g)) and higher wet/dry ratio (Figure 4(h)) in CXCL14-Tg mice. However, the overexpression of PKM2 failed to alter pulmonary expressions of CXCL14 (Figure 4(i)). Together, those results indicated that PKM2 overexpression abolished the protective effects of CXCL14 against VILI in mice.

## 4. Discussion

The major findings of the present study can be summarized as follows: (1) MV induced excessive pulmonary cytokine expressions together with decreased CXCL14 and increased PKM2 expressions. (2) Either CXCL14 overexpression or PKM2 knockdown was able to reduce pulmonary cytokine productions, which subsequently attenuates VILI in mice. (3) CXCL14 overexpression attenuated VILI through the downregulation of PKM2-mediated pulmonary cytokine productions.

Inflammation is a highly reactive response, which is essential for eliminating pathogens and repairing tissue after injury [17]. The release of proinflammatory cytokines has been considered the hallmark of inflammation. Specifically, numerous evidences suggested that pulmonary proinflammatory cytokines (IL-1*β*, IL-6) and other injury response genes are upregulated after MV [18]. These studies showed that these proinflammatory cytokines likely play a vital role in the development of VILI. In the present study, our results were in line with previous findings. In the past decade, the glycolytic regulator PKM2 has demonstrated to promote inflammation through regulation of the release of IL-1*β* [7]. In addition, Zhong and colleagues [19] suggested that glycolytic inhibition could be an effective anti-inflammatory strategy in treating acute lung injury. In this study, we investigated the role of PKM2 in inflammatory response of VILI. We found that mechanical ventilation induced excessive PKM2 expressions. Consistent with previous reports, PKM2 remarkably upregulated pulmonary cytokine expressions.

CXCL14, a recently described non-ELR cytokine, plays putative multiple roles in inflammation and immunity [14]. CXCL14 mRNA is detected in monocytes and B cells following stimulation with LPS [20]. However, some other studies have showed that TNF-*α* and LPS could inhibit the expression of CXCL14 [21, 22]. In our experiments, we found that MV reduced the expressions of pulmonary CXCL14 together with excessive production of cytokines including TNF-*α*, IL-1*β*, and IL-6. Correlation analysis suggested a negative association between pulmonary CXCL14 expression and all these proinflammatory cytokines. Conversely, it demonstrated a positive association between pulmonary PKM2 expression and proinflammatory cytokines. Our work showed that CXCL14 overexpression was able to downregulate pulmonary expression of PKM2 after MV, which subsequently resulted in reduced cytokine productions and improved pulmonary morphology. Importantly, the overexpression of pulmonary PKM2 abolished the protective effects of CXCL14 against VILI together with increased proinflammatory cytokine productions.

One of the important findings of this study is that we firstly found that CXCL14 overexpression inhibits pulmonary PKM2 expression. However, the underlying molecular mechanisms by which CXCL14 overexpression downregulates PKM2 expression have not been investigated in the present study. It has been reported that HIF-1*α*/NF-*κ*B signaling can mediate transcriptional upregulation of the PKM gene [23], which results in increased PKM2 expression. In addition, activated RelA interacts with HIF-1*α*, ultimately binds RelA to the PKM gene promoter, and activates PKM transcription [24]. Lu and colleagues found a negative interaction between CXCL14 and NF-*κ*B activation [25]. In the present study, whether CXCL14 inhibits PKM2 expression through the regulation of NF-*κ*B signaling remains unknown. Another important finding of this study is that CXCL14 overexpression is able to inhibit pulmonary inflammation through the downregulation of PKM2. In our animal MV model, we found that MV induced PKM2 expressions in injured lungs. Therefore, the inhibition of PKM2 expression with chemical compounds can probably attenuate VILI. It has been reported that shikonin, metformin, vitamin K (VK)3/5, and temozolomide (TMZ) show marked inhibitory effects on PKM2 expression [26–29]. Therefore, all these agents have potential value in the protection of lungs from VILI. In fact, previous studies had suggested that shikonin [30], metformin [31], and VK3 [32] protected lungs against sepsis-associated lung injury and/or VILI.

## 5. Conclusion

Our results suggested that the overexpression of CXCL14 attenuates VILI though the downregulation of PKM2-mediated proinflammatory cytokine production in a mouse model of MV.

## Data Availability

The data were available upon request to Dr. Zhao.

## Supplementary Materials

Supplementary Materials Figure S1: transfection of Sh-PKM2 decreased pulmonary PKM2 expressions. PKM2 mRNA and protein expressions were determined by RT-PCR and Western blots at 48 hours after Sh-PKM2 or Sh-Scram transfections. Our data suggested that the transfection of Sh-PKM2 resulted in apparent decreases in pulmonary PKM2 expressions. Figure S2: histological evaluation of lungs after MV. Histological evaluation under light microscope was performed to determine the pulmonary injuries after MV. In the present study, we observed apparent pulmonary edema, neutrophil infiltration, and slight hemorrhage of injured lungs. CXCL14 overexpression attenuates histological damages; however, PKM2 overexpression abolished the protective effects of CXCL14 against VILI.
