# Supplementary material for: Overexpression of CXCL14 Alleviates Ventilator-Induced Lung Injury through the Downregulation of PKM2-Mediated Cytokine Production
Source: Mediators Inflamm. 2020 Jul 23;2020:7650978. doi: 10.1155/2020/7650978 (PMC7396076; doi:10.1155/2020/7650978)
Supplement: Supplementary Materials — Figure S1: transfection of Sh-PKM2 decreased pulmonary PKM2 expressions. PKM2 mRNA and protein expressions were determined by RT-PCR and Western blots at 48 hours after Sh-PKM2 or Sh-Scram transfections. Our data suggested that the transfection of Sh-PKM2 resulted in apparent decreases in pulmonary PKM2 expressions. Figure S2: histological evaluation of lungs after MV. Histological evaluation under light microscope was performed to determine the pulmonary injuries after MV. In the present study, we observed apparent pulmonary edema, neutrophil infiltration, and slight hemorrhage of injured lungs. CXCL14 overexpression attenuates histological damages; however, PKM2 overexpression abolished the protective effects of CXCL14 against VILI. [file 7650978.f1.pdf]

# Supplementary materials

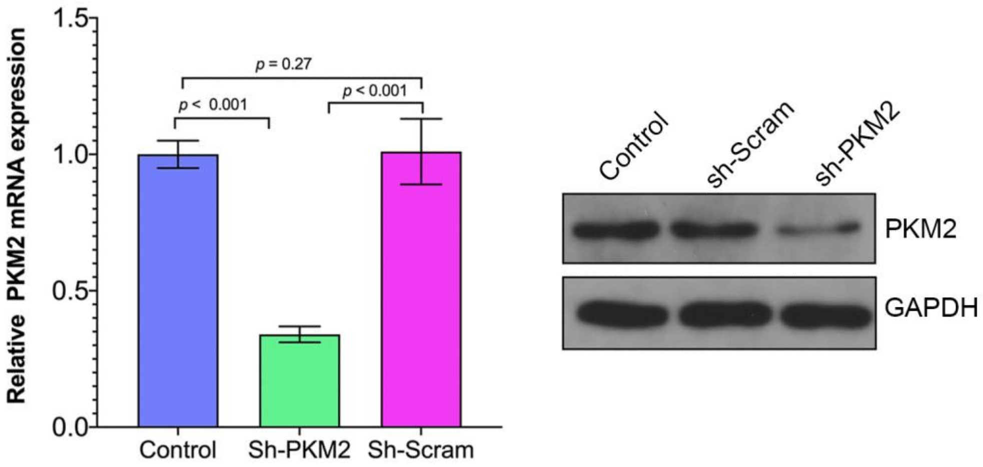


**Figure S1. Transfection of shPKM2 decreased pulmonary PKM2 expressions.** PKM2 mRNA and protein expressions were determined by RT-PCR and western-blots at 48 hours after Sh-PKM2 or Sh-Scram transfections. Our data suggested that the transfection of Sh-PKM2 resulted in apparent decreases in pulmonary PKM2 expressions.

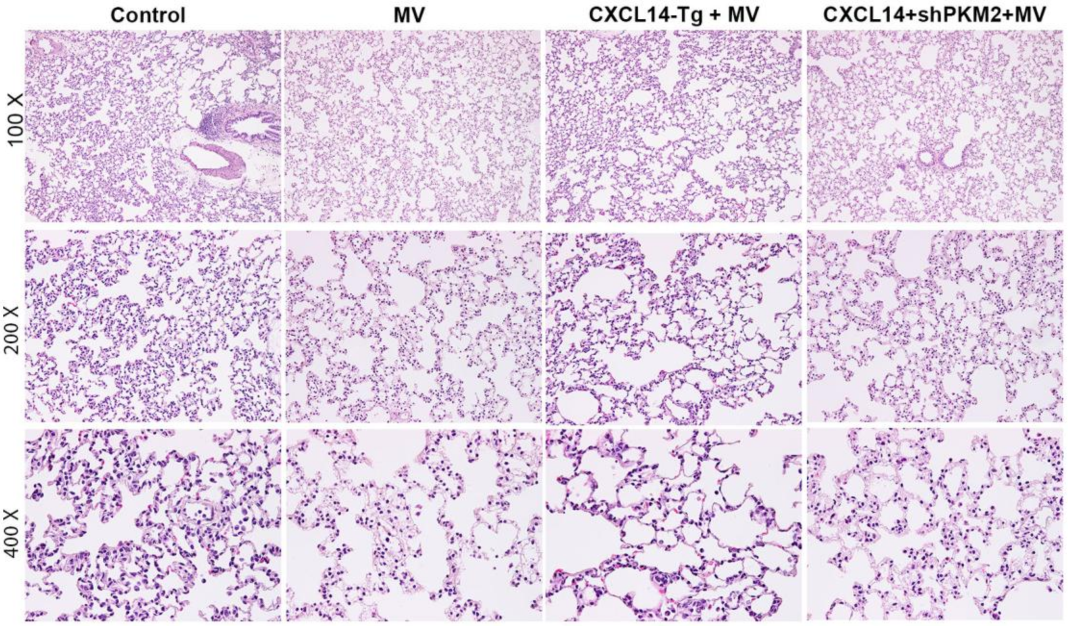


**Figure S2. Histological evaluation of lungs after MV.** Histological evaluation under light microscope were performed to determine the pulmonary injuries after MV. In the present study, we observed apparent pulmonary edema, neutrophil infiltration and slight hemorrhage of injured lungs. CXCL14 overexpression attenuates histological damages; however, PKM2 overexpression abolished the protective effects of CXCL14 against VILI.
